# Measles, mumps and rubella vaccination coverage in capitals and interior region municipalities of Northeast Brazil: a household survey in a cohort of children born in 2017 and 2018

**DOI:** 10.1590/S2237-96222024v33e20231296.especial2.en

**Published:** 2024-12-16

**Authors:** Adjoane Mauricio Silva Maciel, Alberto Novaes Ramos, Anderson Fuentes Ferreira, Taynara Lais Silva, Carla Magda Allan Santos Domingues, Ramon da Costa Saavedra, Jaqueline Caracas Barbosa, Ana Paula França, Ligia Regina Franco Sansigolo Kerr, Maria da Gloria Teixeira, José Cássio de Moraes, ICV 2020 Group, Adriana Ilha da Silva, Alberto Novaes Ramos, Ana Paula França, Andrea de Nazaré Marvão Oliveira, Antonio Fernando Boing, Carla Magda Allan Santos Domingues, Consuelo Silva de Oliveira, Ethel Leonor Noia Maciel, Ione Aquemi Guibu, Isabelle Ribeiro Barbosa Mirabal, Jaqueline Caracas Barbosa, Jaqueline Costa Lima, José Cássio de Moraes, Karin Regina Luhm, Karlla Antonieta Amorim Caetano, Luisa Helena de Oliveira Lima, Maria Bernadete de Cerqueira Antunes, Maria da Gloria Teixeira, Maria Denise de Castro Teixeira, Maria Fernanda de Sousa Oliveira Borges, Rejane Christine de Sousa Queiroz, Ricardo Queiroz Gurgel, Rita Barradas Barata, Roberta Nogueira Calandrini de Azevedo, Sandra Maria do Valle Leone de Oliveira, Sheila Araújo Teles, Silvana Granado Nogueira da Gama, Sotero Serrate Mengue, Taynãna César Simões, Valdir Nascimento, Wildo Navegantes de Araújo

**Affiliations:** 1 Universidade Federal do Ceará, Faculdade de Medicina, Programa de Pós-graduação em Saúde Pública, Fortaleza, Ceará, Brazil; 2 Organização Panamericana da Saúde, Brasília, Distrito Federal, Brazil; 3 Universidade Federal da Bahia, Instituto de Saúde Coletiva, Salvador, Bahia, Brazil; 4 Faculdade de Ciências Médicas da Santa Casa de São Paulo, SP, São Paulo, Brazil; Universidade Federal do Espírito Santo, Vitória, ES, Brazil; Universidade Federal do Ceará, Departamento de Saúde Comunitária, Fortaleza, CE, Brazil; Faculdade Ciências Médicas Santa Casa de São Paulo, São Paulo, SP, Brazil; Secretaria de Estado da Saúde do Amapá, Macapá, AP, Brazil; Universidade Federal de Santa Catarina, SC, Brazil; Organização Pan-Americana da Saúde, Brasília, DF, Brazil; Instituto Evandro Chagas, Belém, PA, Brazil; Faculdade de Ciências Médicas Santa Casa de São Paulo, Departamento de Saúde Coletiva, São Paulo, SP, Brazil; Universidade Federal de Mato Grosso, Cuiabá, MT, Brazil; Universidade Federal do Paraná, Curitiba, PR, Brazil; Universidade Federal de Goiás, Goiânia, GO, Brazil; Universidade Federal do Piauí, Teresina, PI, Brazil; Universidade de Pernambuco, Faculdade de Ciências Médicas, Recife, PE, Brazil; Instituto de Saúde Coletiva, Universidade Federal da Bahia, Salvador, BA, Brazil; Secretaria de Estado da Saúde de Alagoas, Maceió, AL, Brazil; Universidade Federal do Acre, Rio Branco, AC, Brazil; Universidade Federal do Maranhão, Departamento de Saúde Pública, São Luís, MA, Brazil; Universidade Federal de Sergipe, Aracaju, SE, Brazil; Secretaria Municipal de Saúde, Boa Vista, RR, Brazil; Fundação Oswaldo Cruz, Mato Grosso do Sul, Campo Grande, MS, Brazil; Fundação Oswaldo Cruz, Escola Nacional de Saúde Pública Sergio Arouca, Rio de Janeiro, RJ, Brazil; Universidade Federal do Rio Grande do Sul, Porto Alegre, RS, Brazil; Fundação Oswaldo Cruz, Instituto de Pesquisa René Rachou, Belo Horizonte, MG, Brazil; Secretaria de Desenvolvimento Ambiental de Rondônia, Porto Velho, RO, Brazil; Universidade de Brasília, Brasília, DF, Brazil

**Keywords:** Cobertura de vacunación, Sarampión, Vacuna contra el sarampión, parotiditis y la rubéola, Encuestas Epidemiológicas, Vaccination Coverage, Measles, Measles-Mumps-Rubella Vaccine, MMR Vaccine, Health Surveys

## Abstract

**Objective:**

To analyze measles, mumps, and rubella vaccination coverage among children up to 24 months old and factors associated with non-vaccination in a 2017−2018 live birth cohort, in state capitals and large interior region cities in Northeast Brazil.

**Methods:**

Population-based survey analyzing vaccination coverage and sociodemographic factors through logistic regression.

**Results:**

For 12,137 children, vaccination coverage was 79.3% (95%CI 76.5;81.8), and the dropout rate was 10.6%. Association with non-vaccination: socioeconomic stratum A (OR-a 1.29; 95%CI 1.10;1.50), living in the interior region (OR-a 1.22; 95%CI 1.07;1.39), no access to the *Bolsa* *Família* Program (OR-a 1.19, 95%CI 1.05;1.34), family income ≤BRL 1,000 (OR-a 1.17, 95%CI 1.03;1.31), mother not working (OR-a 1.28, 95%CI 1.15;1.42), >1 child per mother (OR-a 1.12, 95%CI 1.08;1.17), and no vaccination card (OR-a 10.69, 95%CI 6.27;18.20).

**Conclusion:**

Low vaccination coverage and a high dropout rate in state capitals and municipalities in the interior region of Northeast Brazil.

## Study contributions

**Main results:**

79.3% vaccination coverage with incomplete vaccination associated with living in higher socioeconomic strata and in interior region municipalities, and children without vaccination records. High dropout rates in the state capitals Natal and João Pessoa.

**Implications for services:**

Recognition of low coverage and factors associated with non-vaccination provides health services with relevant information with a view to planning strategic actions to increase measles, mumps and rubella vaccination coverage.

**Perspectives:**

Low coverage and identification of non-vaccination indicate the importance of targeting actions in the Brazilian National Health System to achieve the targets recommended by the National Immunization Program, for populations in Northeast Brazil.

## RESUMEN

**Objetivo:**

Analizar las coberturas de vacunación contra sarampión, paperas y rubéola en niños de hasta 24 meses de edad y los factores asociados a la no vacunación en una cohorte de nacidos vivos en 2017-2018, en capitales y municipios con gran población del interior del Nordeste brasileño.

**Métodos:**

Encuesta nacional poblacional para analizar cobertura vacunal y factores sociodemográficos por regresión logística.

**Resultados:**

En 12.137 niños, cobertura de vacunación del 79,3% (IC95% 76,5;81,8), tasa de deserción del 10,6%. Asociación con no vacunación: estrato socioeconómico A (OR-a 1,29; IC95% 1,10;1,50), vivir en el campo (OR-a 1,22; IC95% 1,07;1,39), no acceder al Programa Bolsa Familia (OR-a 1,19; IC95% 1,05;1,34), ingreso familiar ≤ R$ 1.000,00 (OR-a 1,17; IC95% 1,03;1,31), madre sin trabajo remunerado (OR-a 1,28; IC95% 1,15;1,42), > 1 hijo por madre (OR-a 1,12; IC95% 1,08;1,17), sin carné de vacunación (OR-a 10,69; IC95% 6,27;18,20).

**Conclusión:**

Baja cobertura y alta tasa de abandono en capitales y municipios del interior del Nordeste.

## INTRODUCTION

Consisting of live attenuated measles, mumps and rubella viruses, the triple viral vaccine (against measles, mumps and rubella - MMR) is available free of charge in Brazil via the National Immunization Program (*Programa Nacional de Imunizações* - PNI), and is recommended for routine vaccination of all children at 12 and 15 months of age, with two doses for people aged up to 29 years and one dose for people up to 59 years old.^1^

Measles remains among the leading causes of death among children, mainly due to its high transmissibility,^2^ with epidemics that have caused around 2.6 million deaths per year worldwide, mainly affecting children ≤ 5 years old.^2^

In 2016, after five years without records of cases, Brazil was granted measles elimination certification. However, due to low vaccination coverage, the virus reemerged, with outbreaks in several states.^2^ The continuing transmission scenario in Brazil led to loss of certification in 2019^3-5^ and enabled identification of flaws in Primary Health Care (PHC) immunization programs.^6^

In 2020, elimination of rubella, a vaccine-preventable disease and its main complication, congenital rubella syndrome, continued to be a goal yet to be achieved, in contexts of low vaccination coverage (70.0%) worldwide.^7^ The Region of the Americas, including Brazil, was declared rubella-free in 2015 by the World Health Organization (WHO).^8^ In Brazil, certification of rubella elimination was achieved through the implementation of strategies with the adoption of the MMR vaccine in a national vaccination campaign, and in follow-up campaigns for children and women of childbearing age. Maintaining the elimination of rubella presents a challenge given low vaccination coverage in Brazil.^9^

Reemergence of other vaccine-preventable diseases, in contexts of reduced vaccination coverage, has been recorded in several countries, with intensification during the course of the COVID-19 pandemic, especially due to the increase in social inequities and restrictions on access to healthcare.^4^

A national ecological study analyzing vaccination coverage of the first dose of MMR vaccine from 2006-2020 in Brazil, according to the Brazilian Municipal Deprivation Index (Índice Brasileiro de Privação de *Municípios*), highlighted a generalized decrease in vaccination coverage, especially among more socially vulnerable people, with a greater interannual decline in the North and Northeast regions of the country,^10^ this being a finding similar to that found in 2021 by the Project for the Recovery of High Vaccination Coverage (*Reconquista das Altas Coberturas Vacinais* - PRCV).^11^

The risk of reemergence of measles and other vaccine-preventable diseases with occurrence of outbreaks and epidemics in Brazil has been highlighted by several studies if consistent control measures are not adopted to achieve the vaccination coverage targets recommended by the PNI, particularly in regions with greater socioeconomic and health access inequality, such as the Northeast.^3,4,10,11^ From this perspective, the objective of this study is to analyze MMR vaccination coverage among children up to 24 months old and factors associated with non-vaccination in a cohort of 2017 and 2018 live births, living in state capitals and large interior region cities in Northeast Brazil. The aim is to recognize key aspects to inform health management and planning.

## METHODS

### Study design

This is a population-based study carried out from September 2020 to March 2022 using a cohort of 2017 and 2018 live births as a reference to identify the vaccination path of children aged 24 months. This study is an excerpt from the “Vaccination Coverage Survey in the capital cities of 26 States, the Federal District and 12 inner region cities of children born alive in 2017−2018 living in urban areas” (*Inquérito de cobertura vacinal nas capitais de 26 Estados, no Distrito Federal e em 12 municípios do interior em crianças nascidas vivas em 2017−2018 residentes em área urbana*). The design followed WHO methodological procedures for compiling vaccination coverage estimates.^12^

### Study sites

The study sites selected included four large cities located in the interior region of Northeast Brazil (Vitória da Conquista [Bahia], Caruaru [Pernambuco], Sobral [Ceará] and Imperatriz [Maranhão]) and the nine state capitals of Northeast Brazil (São Luís [Maranhão], Teresina [Piauí], Fortaleza [Ceará], Natal [Rio Grande do Norte], João Pessoa [Paraíba], Recife [Pernambuco], Maceió [Alagoas], Aracaju [Sergipe], Salvador [Bahia]).

The Northeast region of Brazil covers an area of 1,552,175 km² and is the second most populous of the country’s regions, with 54,657,621 inhabitants in 2022 (26.9% of the Brazilian population), a population density of 35.21 inhabitants/km² and 6.7% of the population (3,635,333) aged between 0 and 4 years old.^13^

### Population

The target population was made up of children born alive in 2017-2018 and residing in the municipalities studied, using the Live Birth Information System (*Sistema de Informação de Nascidos Vivos*) as a data source, containing the child’s nominal data, parents and place of residence.

### Sampling procedure 

The complex sampling process by clusters was based on residence data (interior region municipalities and state capitals) and the use of information on schooling/literacy, the average income of heads of household and the proportion of those earning 20 minimum wages or more, classifying, based on these indicators, the census tracts into four socioeconomic strata: A (high) and B (medium high), representing areas with higher income and literacy, and the opposite, indicated by strata C (medium low) and D (low).^12^

The study sample was determined in three stages:

- Stage 1: composition of socioeconomic strata based on head of family schooling and income, using data from the 2010 demographic census and classified by census tracts (A; B; C; D).13
- Stage 2: composition of clusters with ≥ 56 children based on georeferencing of the addresses of live-born children living in the census tracts.
- Stage 3: random selection for each socioeconomic stratum, from a varied number of clusters.

### Data collection and variables 

The selected team of interviewers underwent specific training and they were monitored by the team of researchers during the data collection stage of the survey.^12^ During fieldwork which involved data collection in households, precautionary norms were followed to prevent transmission of COVID-19.

During home visits, a questionnaire was used to interview mothers/guardians of the children in order to identify sociodemographic characteristics: socioeconomic strata (A, B, C, D), municipality (capital, interior), child has a vaccination card (yes, no), use of private vaccination service (yes, no), *Bolsa Família* Program (yes, no), family income in BRL (≤ 1,000; 1,001-3,000; 3,001-8,000; ≥ 8,001), maternal schooling expressed in years of study (0-8, 9-12, 13-15, ≥ 16), age group in years (< 20, 20-34, ≥ 35), paid job (yes, no), average number of children alive per mother (continuous variable), child’s sex (male, female) and child attends daycare/school (yes, no). The children’s vaccination cards were photographed in order to be able to assess the basic vaccination schedule, including records of doses administered in the private vaccination sector, considering in the latter case at least one dose of any vaccine in this type of service.^12^

### Analysis

Sample weights were used for each household visited by calculating selection probability, with calibration according to population groups.^12^

MMR vaccination coverage and the evolution of this indicator were assessed, including the first and second valid doses received by children up to 24 months old, considering schedule completion with two doses, as recommended by the PNI. Therefore, the last valid doses of the complete schedule were used in order to calculate vaccination coverage, in relation to the total number of live births. Based on the composition of the vaccines used to protect against these diseases in public and private settings, the dates on which MMR vaccine and MMRV vaccine (measles, mumps, rubella and varicella) were administered were analyzed jointly, taking into account the equivalent age group for each dose administered in order to calculate vaccination coverage. First doses of the MMR vaccine administered after the child was over 365 days old and second doses administered at least 30 days after the first dose were defined as valid doses.^1,12,14^

Weighted estimates of vaccination coverage with weights and respective 95% confidence intervals (95%CI) were calculated for both doses, for each dose and for the MMR schedule, according to socioeconomic strata and municipalities (state capitals and interior region cities). The criterion for statistical significance was based on a p value < 0.05.^12^

In order to analyze risk factors for incomplete vaccination against MMR (children who did not receive all doses), the adjusted odds ratios (OR-a) with respective 95%CI, for use in the logistic regression were incorporated into the models. In the simple logistic regression model, the categories of variables with a p value < 0.20 were included in the analysis model using the stepwise method, verifying the independent joint effect for the occurrence of incomplete vaccination and the existence of collinearity between variables due to variance inflation factor analysis. The dependent variable, namely vaccination status, took into account the two valid doses of the MMR vaccine, being dichotomized into incomplete vaccination (“vaccination incompleteness”) or full vaccination (“full dose schedule”). 

Three categories were defined for valid doses:

- No record of doses (child did not receive any vaccine dose)
- Incomplete dose schedule (children received one vaccine dose)
- Full dose schedule (child received both vaccine doses)

Incomplete vaccination status was made up of the categories “No dose record” and “Incomplete dose schedule”. The “Full dose schedule” category was taken to indicate compliant vaccination.

The dropout rate of the second dose in relation to the first dose was also evaluated ([percentage of first dose – percentage of second dose]/percentage of first dose).

The data were presented according to socioeconomic stratum, state capitals and interior region cities. STATA version 17 (StataCorp LLC, College Station, TX) was used for the statistical analysis.

### Ethical considerations

The study was approved by the Research Ethics Committee of the *Instituto de Saúde Coletiva da Universidade Federal da Bahia*, as per Opinion No. 3.366.818, on June 4, 2019, and Certificate of Submission for Ethical Appraisal (*Certificado de Apresentação de Apreciação* Ética - CAAE) No. 4306919.5.0000.5030; and by the Research Ethics Committee of the *Irmandade da Santa Casa de São Paulo*, as per Opinion No. 4.380.019, on November 4, 2020, and CAAE No. 39412020.0.0000.5479.

## RESULTS

**Figure 1:**
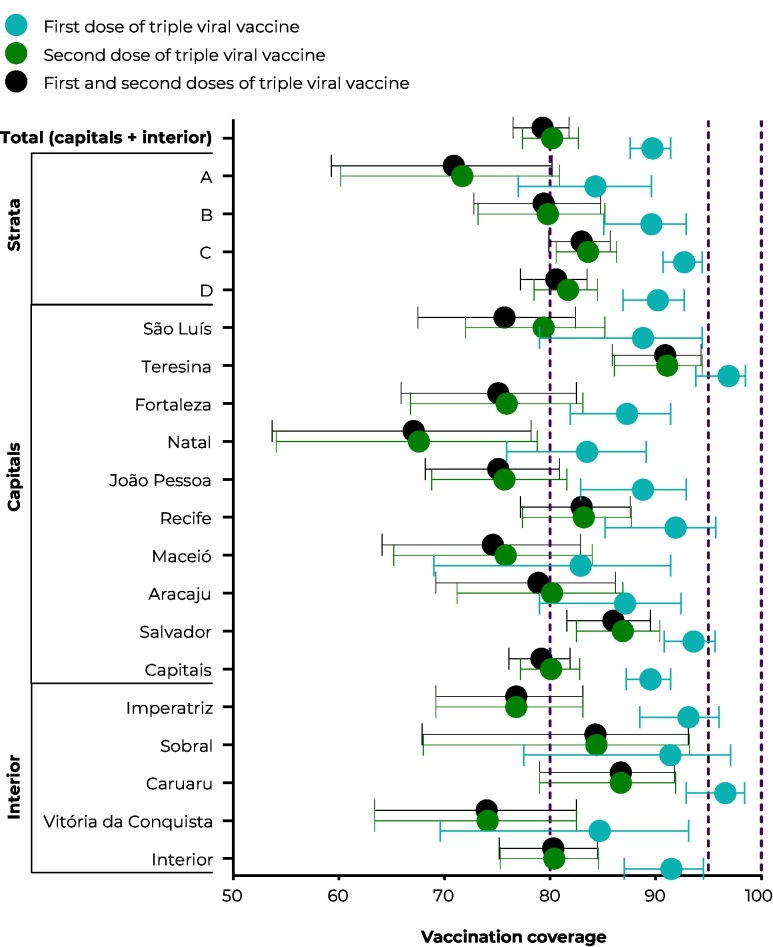
Vaccination coverage of first and second doses, and full coverage of measles, mumps and rubella vaccine in a cohort of 2017 and 2018 live births in state capitals e interior region cities of Northeast Brazil, by socioeconomic strata (n = 12,137)

Among the sample of 12,137 live births in 2017-2018 (Table 1), the highest MMR vaccination coverage was found for the first dose (89.7%, 95%CI 87.6;91.4), mainly in stratum C (92.7%, 95%CI 90.7;94.4). Among the state capitals, the best (full) vaccination coverage was found in Teresina (90.9%, 95%CI 85.9;94.3), while Natal had the poorest vaccination coverage (67.1%, 95%CI 53.7;78.2). Vitória da Conquista had the lowest full vaccination coverage among the interior region cities (74.0%, 95%CI 63.4;82.5) (Figure 1). A sample loss of 525 live births (4.8%) was estimated^13^ for the state capitals, while there were no losses in the interior region cities.

**Table 1:** Absolute and relative frequency of 2017 and 2018 live births in state capital cities and large interior region cities of Northeast Brazil, by socioeconomic strata (n = 12.137)

Variables/socioeconomic strata	A	B	C	D	Total
	**n (%)**	**n (%)**	**n (%)**	**n (%)**	**n (%)**
**Total**	2.701 (22.3)	3.118 (25.7)	3.145 (25.9)	3.173 (26.1)	12.137 (100.0)
**State capitals**					
São Luís	182 (6.7)	223 (7.2)	224 (7.1)	225 (7.1)	854 (7.0)
Teresina	227 (8.4)	225 (7.2)	222 (7.1)	225 (7.1)	899 (7.4)
Fortaleza	312 (11.6)	432 (13.9)	423 (13.4)	445 (14.0)	1,612 (13.3)
Natal	84 (3.1)	153 (4.9)	223 (7.1)	225 (7.1)	685 (5.6)
João Pessoa	226 (8.4)	225 (7.2)	226 (7.2)	227 (7.2)	904 (7.4)
Recife	330 (12.2)	447 (14.3)	462 (14.7)	450 (14.2)	1,689 (13.9)
Maceió	205 (7.6)	279 (8.9)	219 (7.0)	226 (7.1)	929 (7.7)
Aracaju	233 (8.6)	219 (7.0)	222 (7.1)	226 (7.1)	900 (7.4)
Salvador	450 (16.7)	456 (14.6)	456 (14.5)	456 (14.4)	1,818 (15.0)
**Interior region cities**					
Imperatriz	120 (4.4)	113 (3.6)	118 (3.8)	114 (3.6)	465 (3.8)
Sobral	103 (3.8)	119 (3.8)	120 (3.8)	123 (3.9)	465 (3.8)
Caruaru	113 (4.2)	114 (3.7)	116 (3.7)	119 (3.8)	462 (3.8)
Vitória da Conquista	116 (4.3)	113 (3.6)	114 (3.6)	112 (3.5)	455 (3.7)

In the household interviews, 36.0% of mothers/guardians reported receiving the *Bolsa Família* Program benefit, most frequently in stratum D (49.9%). Family income ≤ BRL 1,000 was reported by 38.0% of families (Table 2).

**Table 2:** Family, maternal and child sociodemographic characteristics (%) and 95% confidence intervals (95%CI), in a cohort of 2017 and 2018live births in state capital cities and large interior region cities of Northeast Brazil, by socioeconomic strata (n = 12.137)

Variables/socioeconomic strata	A	B	C	D	Total
	**% (95%CI)**	**% (95%CI)**	**% (95%CI)**	**% (95%CI)**	**% (95%CI)**
**Family characteristics**					
**Bolsa Família Program (yes)**	7.9 (5.7;11.0)	20.1 (15.7;25.4)	37.8 (33.7;42.1)	49.9 (45.5;54.3)	36.0 (33.4;38.7)
**Monthly family income (BRL)**					
≤ 1,000	5.7 (3.8;8.3)	19.1 (15.3;23.6)	37.3 (32.5;42.2)	55.5 (51.1;59.9)	38.0 (35.0;41.2)
1,001-3,000	12.1 (8.6;16.6)	30.8 (25.5;36.7)	44.1 (39.4;49.0)	35.0 (30.5;39.8)	32.5 (29.7;35.5)
3,001-8,000	27.5 (20.6;35.8)	24.6 (19.7;30.4)	14.8 (10.4;20.6)	3.4 (2.5;4.7)	12.9 (10.9;15.3)
≥ 8,001	35.3 (26.3;45.5)	11.9 (6.0;22.2)	1.1 (0.7;1.8)	0.3 (0.0;0.9)	8.0 (5.9;10.8)
Unable to answer/did not answer	19.4 (11.1;31.9)	13.6 (8.3;21.5)	2.8 (1.9;4.1)	5.8 (3.6;9.3)	8.5 (6.3;11.4)
**Maternal characteristics**					
**Age group when child born (years)**					
< 20	1.0 (0.6;1.9)	1.1 (0.7;1.8)	2.5 (1.8;3.6)	4.5 (3.3;6.2)	3.0 (2.4;3.9)
20-34	44.8 (37;52.8)	50.0 (44.2;55.9)	67.9 (64.5;71.2)	65.1 (61.3;68.7)	60.2 (57.5;62.8)
≥ 35	53.9 (45.8;61.8)	48.3 (42.2;54.4)	29.3 (26.1;32.6)	30.0 (26.1;34.3)	36.4 (33.6;39.3)
Unable to answer/did not answer	0.3 (0.2;0.6)	0.6 (0.3;1.0)	0.3 (0.0;0.8)	0.4 (0.2;1.0)	0.4 (0.2;0.6)
**Schooling (years of study)**					
0-8	2.1 (1.3;3.1)	6.3 (4.6;8.7)	8.3 (6.5;10.5)	15.8 (13.4;18.6)	10.5 (9.3;11.9)
9-12	4.8 (2.9;7.7)	9.9 (7.5;12.9)	18.0 (14.8;21.8)	22.1 (19;25.6)	16.6 (14.8;18.5)
13-15	28.0 (21.9;35.1)	33.0 (26.4;40.2)	54.1 (50.3;57.9)	49.4 (45.2;53.5)	44.5 (41.7;47.4)
≥ 16	61.9 (54.2;69.0)	47.6 (38.7;56.6)	17.1 (14.1;20.6)	10.1 (5.5;18.0)	25.6 (22.0;29.7)
Unable to answer/did not answer	3.3 (1.6;7.1)	3.3 (1.8;5.8)	2.5 (1.6;3.8)	2.6 (1.8;3.7)	2.8 (2.2;3.6)
**Paid job (yes)**	68.6 (60.8;75.4)	56.2 (50.8;61.4)	43.0 (39.5;46.5)	36.4 (33.0;39.9)	**46.0 (43.5;48.7)**
**Average of child alive per mother**	1.91 (1.87;1.95)	1.99 (1.95;2.03)	2.03 (1.99;2.07)	2.21 (2.17;2.26)	**2.04 (2.02;2.06)**
**Children’s characteristics**					
**Child’s sex**					
Male	50.4 (43.0;57.7)	51.4 (46.6;56.1)	51.6 (47.5;55.7)	50.7 (47.8;53.6)	50.9 (48.8;53.1)
Female	49.6 (42.3;57.0)	48.6 (43.9;53.4)	48.4 (44.3;52.5)	49.3 (46.4;52.2)	49.1 (46.9;51.2)
**Has a vaccination card (yes)**	98.9 (95.9;99.7)	99.3 (98.7;99.6)	99.1 (98.2;99.6)	99.0 (97.2;99.6)	**99.0 (98.3;99.4)**
**Use of private service for vaccination (yes)**	52.2 (43;61.3)	26.0 (19.1;34.3)	7.8 (6.0;10.1)	5.7 (2.2;13.8)	**16.9 (13.6;20.8)**
**Attends daycare/school (yes)**	48.7 (37.9;59.6)	37.6 (29.9;45.9)	34.4 (29.8;39.4)	31.1 (26.7;35.8)	**35.7 (32.4;39.1)**

The majority of the children’s mothers were between 20 and 34 years old (60.2%), had schooling comprised of between 13 and 15 years of study (44.5%) and paid employment (46.0%), with an average of 2.04 children per mother (Table 2).

The children were more frequently of the male sex (50.9%), 99.0% of the sample had vaccination cards, with no difference between strata. Use of a private vaccination service was found for 16.9% of children, with greater frequency in stratum A (52.2%), and 35.7% of the sample attended daycare/school, with lower frequency in stratum D (31.1%) (Table 2).

Association was identified between non-vaccination against MMR, considering the full vaccination schedule, with the following sociodemographic characteristics: living in areas corresponding to stratum A (OR-a 1.29, 95%CI 1.10;1.50), living in the interior region (OR-a 1.22, 95%CI 1.07;1.39), not having access to the *Bolsa Família* Program (OR-a 1.19, 95%CI 1.05;1.34), family income ≤ BRL 1,000 (OR-a 1.17, 95%CI 1.03;1.31), mother not having a paid job (OR-a 1.28, 95%CI 1.15;1.42), having more than one live child per mother (OR-a 1.12, 95%CI 1.08;1.17) and not having a vaccination card (OR-a 10.69, 95%CI 6.27;18.20 ) (Table 3).

**Table 3:** Crude and adjusted odds ratio (OR) and 95% confidence intervals (95%CI) of incomplete vaccination and non-vaccination against measles, mumps and rubella by sociodemographic factors of children born in 2017 and 2018, residing in capital cities and municipalities with a large population in the interior of Northeast Brazil (n=12,137)

Variables	Crude OR (95%CI)	p-value	Adjusted OR (95%CI)	p-value
**Socioeconomic strata**				
A	1.42 (1.25;1.61)	0.138	1.29 (1.10;1.50)	0.057
B	1.35 (1.19;1.53)		1.27 (1.11;1.46)	
C	1.00		1.00	
D	1.19 (1.05;1.35)		1.15 (1.00;1.32)	
**Municipality**				
Capital	1.00	0.012	1.00	0.001
Interior	1.17 (1.03;1.31)		1.22 (1.07;1.39)	
**Family characteristics**				
*Bolsa* *Família* **Program**				
Yes	1.00	< 0.001	1.00	0.014
No	1.20 (1.10;1.32)		1.19 (1.05;1.34)	
**Monthly family income (BRL)**				
≤ 1,000	1.06 (0.95;1.18)	0.026	1.17 (1.03;1.31)	0.300
1,001-3,000	1.00		1.00	
3,001-8,000	1.15 (1.00;1.32)		1.03 (0.87;1.21)	
≥ 8,001	1.15 (0.97;1.37)		0.92 (0.73;1.15)	
**Maternal characteristics**				
**Age group when child born (years)**				
< 20	1.00	0.860	–	–
20-34	1.30 (0.98;1.73)		–	
≥ 35	1.26 (0.95;1.68)		–	
**Schooling (years of study)**				
0-8	1.00	0.571	–	–
9-12	1.10 (0.93;1.29)		–	
13-15	0.99 (0.85;1.14)		–	
≥ 16	1.10 (0.94;1.28)		–	
**Maternal paid job**				
Yes	1.00	< 0.001	1.00	< 0.001
No	1.27 (1.16;1.39)		1.28 (1.15;1.42)	
**Children alive**				
Average of more than one child alive per mother	1.08 (1.04;1.12)	< 0.001	1.12 (1.08;1.17)	< 0.001
**Children’s characteristics**				
**Child’s sex**				
Male	1.00	0.278	–	–
Female	1.05 (0.96;1.15)		–	
**Has a vaccination card**				
Yes	1.00	< 0.001	1.00	< 0.001
No	10.93 (6.96;17.15)		10.69 (6.27;18.20)	
**Use of private service for vaccination**				
Yes	1.24 (1.10;1.40)	0.001	1.12 (0.95;1.33)	0.310
No	1.00		1.00	
**Attends daycare/school**				
Yes	1.00	0.021	1.00	0.179
No	1.11 (1.02;1.22)		1.08 (0.97;1.19)	

In the analysis of dose completeness patterns (no dose record, incomplete dose schedule and full dose schedule), full vaccination coverage with two valid MMR vaccine doses accounted for 79.3% (95%CI 76.5;81.8). There was a higher and lower proportion, respectively, in stratum C (83.0%, 95%CI 79.9;85.7) and stratum A (70.9%, 95%CI 59.3;80.2). 9.5% (95%CI 7.8;11.4) of the children (n = 1,179) had no record of receiving any MMR vaccine dose and 11.3% (95%CI 9.8;12.9) of the children (n = 1,365) had not completed the vaccination schedule. Stratum A accounted for the highest proportion of children with no record of administered doses (14.9%, 95%CI 9.8;22.1) and with an incomplete dose schedule (14.2%, 95%CI 9. 2;21,3). With regard to the state capitals, Natal had the highest proportion of children with no administered doses (15.9%, 95%CI 10.4;23.7) as well as incomplete doses (16.9%, 95%CI 10.8 ;25.7). In the interior region cities, Vitória da Conquista stood out with the highest proportion of children with no record of administered MMR vaccine doses (15.2%, 95%CI 6.9;30.3) (Table 4).

**Table 4:** Proportion in (%) and 95% confidence intervals (95%CI) of measles, mumps and rubella non-vaccination, incomplete vaccination, vaccine coverage, and dropout rate in a 2017 and 2018 live birth cohort in state capitals and large cities in the interior region of Northeast Brazil, by socioeconomic strata (n = 12,137)

Variables	Non-vaccination (no record of doses)	Incomplete vaccination (incomplete dose schedule)	Vaccination coverage (full dose schedule)	Dropout rate
	**% (95%CI)**	**% (95%CI)**	**% (95%CI)**	**%**
**Total**	9.5 (7.8;11.4)	11.3 (9.8;12.9)	79.3 (76.5;81.8)	10.6
**Socioeconomic strata**				
A	14.9 (9.8;22.1)	14.2 (9.2;21.3)	70.9 (59.3;80.2)	15.0
B	10.0 (6.8;14.5)	10.6 (8.0;13.9)	79.4 (72.8;84.8)	10.9
C	6.6 (5.1;8.6)	10.4 (8.3;13.0)	83.0 (79.9;85.7)	9.8
D	8.7 (6.4;11.7)	10.8 (9.1;12.8)	80.6 (77.2;83.5)	9.4
**State capitals**				
São Luís	7.6 (4.1;13.7)	16.7 (11.7;23.2)	75.7 (67.5;82.4)	10.6
Teresina	2.9 (1.4;6.1)	6.2 (3.4;10.8)	90.9 (85.9;94.3)	5.9
Fortaleza	11.9 (8.1;17.2)	13.0 (9.1;18.2)	75.1 (65.9;82.5)	13.2
Natal	15.9 (10.4;23.7)	16.9 (10.8;25.7)	67.1 (53.7;78.2)	19.0
João Pessoa	10.5 (6.5;16.6)	14.4 (11.5;17.7)	75.1 (68.2;80.9)	14.7
Recife	7.9 (4.1;14.7)	9.1 (6.9;11.9)	83.0 (77.2;87.6)	9.5
Maceió	15.9 (7.7;30.0)	9.4 (6.4;13.6)	74.6 (64.1;82.9)	8.6
Aracaju	11.6 (6.8;19.3)	9.5 (5.3;16.3)	78.9 (69.2;86.2)	8.0
Salvador	5.4 (3.7;7.9)	8.6 (5.9;12.4)	86.0 (81.6;89.5)	7.1
State capitals	9.6 (7.8;11.7)	11.3 (9.7;13.0)	79.2 (76.1;81.9)	10.4
**Interior region cities**				
Imperatriz	6.9 (4.0;11.5)	16.3 (10.2;24.9)	76.8 (69.2;83.1)	17.5
Sobral	8.5 (2.9;22.5)	7.3 (4.0;13.0)	84.3 (67.9;93.1)	7.7
Caruaru	3.4 (1.6;7.1)	10.0 (6.2;15.6)	86.7 (79.0;91.8)	10.2
Vitória da Conquista	15.2 (6.9;30.3)	10.8 (6.6;17.2)	74.0 (63.4;82.5)	12.5
Interior	8.4 (5.4;12.9)	11.3 (8.7;14.6)	80.3 (75.2;84.5)	12.2

The overall dropout rate for the second MMR dose was 10.6%, with 15.0% in stratum A. The municipalities with the highest dropout rate proportions were: Natal (19.0%) and João Pessoa (14.7%) among the state capitals, and Imperatriz (17.5%) among the interior region cities of the Northeast (Table 4).

## DISCUSSION

This study confirms the worrying scenario of low MMR vaccination coverage without achieving the full vaccination schedule in more than a fifth of children up to 24 months old in the state capitals and in four large interior region cities in Northeast Brazil, in the period from 2017-2018. Worthy of note is the critical fact that 10% of the child population surveyed did not have a record of receiving any MMR vaccine dose on their vaccination cards. The MMR vaccination coverage target stipulated by the PNI (95%) was not achieved in any state capital or interior region city in the Northeast,^15,16^ with the lowest vaccination coverage being found for Natal and Vitória da Conquista, respectively. Children living in areas corresponding to the highest socioeconomic stratum and in interior region cities, family income ≤ BRL 1,000, no record of vaccination, mothers without a paid job, mothers with more than one child, child not attending school/daycare and family without access to the *Bolsa Família* Program were conditions associated with incomplete vaccination and/or non-vaccination against MMR in municipalities in Northeast Brazil. The increased susceptibility to measles of a considerable proportion of children in these cohorts also extends to mumps and rubella.

This is a scenario that reflects the increase in susceptibility and continuing risk of transmission of vaccine-preventable diseases^17^ and reiterates the different dimensions of social vulnerability, therefore demanding more active innovative strategies to intensify vaccination actions in the Brazilian National Health System (*Sistema Único de Saúde* - SUS).^10^

Association between incomplete vaccination and/or non-vaccination against MMR and living in large interior region cities in the Northeast reveals, in an even more striking way, possible barriers to access to vaccination in PHC in addition to social and health inequities.^10^ It is noteworthy that the shortcomings of PHC actions in guaranteeing population coverage was associated with incomplete vaccination in the Northeast region of Brazil in several studies.^4,18,19^

These public health shortcomings in interior region municipalities include issues related to rural contexts^13^ where important problems still persist in achieving vaccination coverage and quality in general.^18^ Vaccination coverage in these rural realities was found to be around 1.5 times lower than in urban areas. Likewise, living in strata with better socioeconomic conditions in the state capitals and interior region municipalities surveyed in the Northeast was significantly associated with incomplete vaccination and/or non-vaccination against MMR. This finding may possibly be associated with issues related to vaccination hesitancy, demonstrated by the decision of parents/guardians to postpone and/or refuse to vaccinate their children,^19^ a fact more commonly seen in these populations.^14^ Another possible explanation is greater access to services not providing vaccination, which may have contributed to the failure to complete the full schedule, possibly due to the inadequacy of monitoring the vaccination status by the public health service, in addition to communication with private services about vaccination strategies and timely vaccination data.^19^

Low MMR vaccination coverage previously evidenced by other national surveys in Brazil^19^ currently persists, made worse by high dropout rates among interior region cities and state capitals in the Northeast,^14^ this being a fact that contributes to the risk of reemergence and/or recurrence of cases, especially among more socially vulnerable populations, usually more affected by vaccine-preventable diseases and with more serious clinical syndromes.^16,18^

Although studies demonstrate the decline in vaccination coverage occurring throughout Brazil, with only half of the municipalities achieving the target recommended by the PNI, socioeconomic disparities portrayed in the Northeast region point to the occurrence of this problem in an even more accentuated manner, demonstrated beyond lower vaccination coverage, given the greater annual drops in MMR vaccination.^10^

In 2015 and 2016, the Region of the Americas was declared an area free of rubella and measles transmission. However, in the year of the most recent cohort (2018), in the North and Northeast regions of Brazil, there was, respectively, reemergence of the measles virus and higher incidence of the disease in Brazil.^20^ This is a critical epidemiological context given the low and heterogeneous MMR vaccination coverage in Brazil^4,21,22^ and the high migratory flow between countries with disease transmission in Latin America. Brazil lost its certification of measles elimination in 2019, after a year of continuous transmission of the disease.^4^ Moreover, maintaining the elimination of rubella^7,8^ remains a challenge in this scenario of low vaccination coverage.^14^

Health inequities intensified by the COVID-19 pandemic in 2020,^23^ in addition reduced access to SUS health services, contributed to reduced vaccination actions, these being facts that, together, explain the continuing and even worsening not only of low measles vaccination coverage, but also that of mumps and rubella, in Brazil.^4,24^

In these scenarios, it is imperative to highlight the important role of PHC as a gateway and coordinator of care within the scope of the SUS in promoting vaccination, evaluation, monitoring and active household follow-up actions to guarantee that the vaccination schedule is kept up to date. Monitoring and evaluation of vaccination actions underway, as well as dissemination of information on vaccination status, are strategic measures for decision-making within vaccination coverage surveillance, in addition to being important evidence for guiding the achievement of the PNI targets.^25^

The reduction in MMR vaccination coverage in the last decade led to the continuing high risk of reemergence of cases in Brazil, even though it is a fundamental action for control. Information, education and communication actions, qualification of surveillance integrated into PHC, recognition of access barriers for vaccination equity and strengthening the PNI with increased availability of vaccines and health professionals are essential for the recovery of high vaccination coverage and its homogeneity.^4,19,21,26^

As the most common benefit among families in the stratum with the poorest socioeconomic conditions, the *Bolsa Família* Program is an important income transfer policy in Brazil, contributing decisively to greater vaccination adherence. This is a strategic condition stipulated by the Program, as it requires the full vaccination schedule to be kept up to date in order to continue receiving the benefit and is, therefore, an effective measure for improving vaccination coverage, including MMR vaccination coverage.^27^

On the other hand, strategic planning in PHC territories is fundamental and cost-effective, even given the high complexity and cost of its execution in different Brazilian realities.^24^ Actions must also bring into perspective more efficient approaches, focusing on local challenges, aligned with the Immunization Agenda 2030,^6^ as is the case of the PRCV, developed in an agreed and shared manner, based on local health systems, in PHC territories, with broad social participation.^11^

The limitations of this study include the use of data from the 2010 demographic census for stratification and selection of census tracts and forming the sample in each state surveyed. The restriction of researchers’ access to households, especially in the higher socioeconomic strata and due to the occurrence of the COVID-19 pandemic, led to data collection limitations in some households, particularly due to the population’s fear related to transmission of the disease. The low quality of photographs of some vaccination cards, in addition to the lack of standards for recording doses and vaccines administered by public and private vaccination services, may have led to errors in the recording of data on the vaccination cards. In order to minimize these errors, training and supervision were provided for the data collection team and for the team that input the information collected from vaccination records. Even with these limitations, this study provides important evidence for a better understanding of the critical scenario of low measles vaccination coverage and, therefore, increased risk of transmission of vaccine-preventable diseases, including mumps and rubella. We highlight the importance of having a greater understanding of limitations related to socioeconomic conditions and access to health services, in order to implement even more efficient and feasible strategies for expanding access to vaccination actions in PHC territories.

In conclusion, low MMR vaccination coverage and a high percentage of non-vaccination against MMR among children up to 24 months old in state capitals and interior region cities in Northeast Brazil have been confirmed by this study. It is worth highlighting the low full vaccination coverage of the MMR scheme, especially the second dose. The drop in this percentage in the 2017-2018 live birth cohort reinforces the persistence and expansion of the problem, in addition to a critical scenario of non-vaccination, with more than a thousand children not having received any dose of MMR vaccine, in different contexts of social inequities.
