# Supplementary material for: Measles, mumps and rubella vaccination coverage in capitals and interior region municipalities of Northeast Brazil: a household survey in a cohort of children born in 2017 and 2018
Source: Epidemiol Serv Saude. 2024 Dec 16;33(spe2):e20231296. doi: 10.1590/S2237-96222024v33e20231296.especial2.en (PMC11654816; doi:10.1590/S2237-96222024v33e20231296.especial2.en)
Supplement: Translation

# Translation: Cobertura da vacina contra sarampo, caxumba e rubéola em capitais e municípios do interior do Nordeste brasileiro: inquérito domiciliar em coorte de crianças nascidas em 2017 e 2018

**DOI:** 10.1590/S2237-96222024v33e20231296.especial2.pt

**Authors:** Adjoane Mauricio Silva Maciel, Alberto Novaes Ramos, Anderson Fuentes Ferreira, Taynara Lais Silva, Carla Magda Allan Santos Domingues, Ramon da Costa Saavedra, Jaqueline Caracas Barbosa, Ana Paula França, Ligia Regina Franco Sansigolo Kerr, Maria da Gloria Teixeira, José Cássio de Moraes, Grupo ICV 2020, Adriana Ilha da Silva, Alberto Novaes Ramos, Ana Paula França, Andrea de Nazaré Marvão Oliveira, Antonio Fernando Boing, Carla Magda Allan Santos Domingues, Consuelo Silva de Oliveira, Ethel Leonor Noia Maciel, Ione Aquemi Guibu, Isabelle Ribeiro Barbosa Mirabal, Jaqueline Caracas Barbosa, Jaqueline Costa Lima, José Cássio de Moraes, Karin Regina Luhm, Karlla Antonieta Amorim Caetano, Luisa Helena de Oliveira Lima, Maria Bernadete de Cerqueira Antunes, Maria da Gloria Teixeira, Maria Denise de Castro Teixeira, Maria Fernanda de Sousa Oliveira Borges, Rejane Christine de Sousa Queiroz, Ricardo Queiroz Gurgel, Rita Barradas Barata, Roberta Nogueira Calandrini de Azevedo, Sandra Maria do Valle Leone de Oliveira, Sheila Araújo Teles, Silvana Granado Nogueira da Gama, Sotero Serrate Mengue, Taynãna César Simões, Valdir Nascimento, Wildo Navegantes de Araújo

**Affiliations:** 1 Universidade Federal do Ceará, Faculdade de Medicina, Fortaleza, Ceará, Brasil; 2 Organização Panamericana da Saúde, Brasília, Distrito Federal, Brasil; 3 Universidade Federal da Bahia, Instituto de Saúde Coletiva, Salvador, Bahia, Brasil; 4 Faculdade de Ciências Médicas da Santa Casa de São Paulo, SP, São Paulo, Brasil; Universidade Federal do Espírito Santo, Vitória, ES, Brasil; Universidade Federal do Ceará, Departamento de Saúde Comunitária, Fortaleza, CE, Brasil; Faculdade Ciências Médicas Santa Casa de São Paulo, São Paulo, SP, Brasil; Secretaria de Estado da Saúde do Amapá, Macapá, AP, Brasil; Universidade Federal de Santa Catarina, SC, Brasil; Organização Pan-Americana da Saúde, Brasília, DF, Brasil; Instituto Evandro Chagas, Belém, PA, Brasil; Faculdade de Ciências Médicas Santa Casa de São Paulo, Departamento de Saúde Coletiva, São Paulo, SP, Brasil; Universidade Federal de Mato Grosso, Cuiabá, MT, Brasil; Universidade Federal do Paraná, Curitiba, PR, Brasil; Universidade Federal de Goiás, Goiânia, GO, Brasil; Universidade Federal do Piauí, Teresina, PI, Brasil; Universidade de Pernambuco, Faculdade de Ciências Médicas, Recife, PE, Brasil; Instituto de Saúde Coletiva, Universidade Federal da Bahia, Salvador, BA, Brasil; Secretaria de Estado da Saúde de Alagoas, Maceió, AL, Brasil; Universidade Federal do Acre, Rio Branco, AC, Brasil; Universidade Federal do Maranhão, Departamento de Saúde Pública, São Luís, MA, Brasil; Universidade Federal de Sergipe, Aracaju, SE, Brasil; Secretaria Municipal de Saúde, Boa Vista, RR, Brasil; Fundação Oswaldo Cruz, Mato Grosso do Sul, Campo Grande, MS, Brasil; Fundação Oswaldo Cruz, Escola Nacional de Saúde Pública Sergio Arouca, Rio de Janeiro, RJ, Brasil; Universidade Federal do Rio Grande do Sul, Porto Alegre, RS, Brasil; Fundação Oswaldo Cruz, Instituto de Pesquisa René Rachou, Belo Horizonte, MG, Brasil; Secretaria de Desenvolvimento Ambiental de Rondônia, Porto Velho, RO, Brasil; Universidade de Brasília, Brasília, DF, Brasil

**Keywords:** Cobertura Vacinal, Sarampo, Vacina contra Sarampo-Caxumba-Rubéola, Inquéritos Epidemiológicos

## Abstract

**Objetivo:**

Analisar a cobertura vacinal contra sarampo, caxumba e rubéola em crianças até 24 meses de idade e os fatores associados à não vacinação em coorte de nascidos-vivos em 2017-2018, em capitais e municípios de grande porte populacional do interior do Nordeste brasileiro.

**Métodos:**

Inquérito domiciliar populacional com análise de cobertura vacinal e fatores sociodemográficos por regressão logística.

**Resultados:**

Em 12.137 crianças, cobertura vacinal de 79,3% (IC_95_% 76,5;81,8) e taxa de abandono de 10,6%. Associação à não vacinação: estrato socioeconômico A (OR-a 1,29; IC_95_% 1,10;1,50), residir no interior (OR-a 1,22; IC_95_% 1,07;1,39), não acesso ao Programa Bolsa Família (OR-a 1,19; IC_95_% 1,05;1,34), renda familiar ≤ R$ 1.000,00 (OR-a 1,17; IC_95_% 1,03;1,31), mãe sem trabalho remunerado (OR-a 1,28; IC_95_% 1,15;1,42), > 1 filho por mãe (OR-a 1,12; IC_95_% 1,08;1,17), sem caderneta de vacinação (OR-a 10,69; IC_95_% 6,27;18,20).

**Conclusão:**

Baixa cobertura e alta taxa de abandono vacinal em capitais e municípios do interior do Nordeste.

## Contribuições do estudo

**Principais resultados:**

Cobertura vacinal de 79,3% com vacinação incompleta associada a residir em estratos socioeconômicos mais altos e em municípios do interior, e a crianças sem caderneta de vacinação. Altas taxas de abandono nos municípios de Natal e João Pessoa.

**Implicações para os serviços:**

O reconhecimento da baixa cobertura e fatores associados à não vacinação oportuniza aos serviços de saúde informações relevantes com vistas ao planejamento de ações estratégicas para o aumento da cobertura vacinal contra sarampo, caxumba e rubéola.

**Perspectivas:**

A baixa cobertura e identificação da não vacinação indicam a importância do direcionamento das ações no Sistema Único de Saúde para o alcance das metas preconizadas pelo Programa Nacional de Imunizações, para populações na região Nordeste do Brasil.

## INTRODUÇÃO

Constituída por vírus vivos atenuados de sarampo, caxumba e rubéola, a vacina tríplice viral (contra sarampo, caxumba e rubéola - SCR) está disponível gratuitamente no Brasil via Programa Nacional de Imunizações (PNI), sendo recomendada para vacinação de rotina de todas as crianças aos 12 e 15 meses de idade, com duas doses para pessoas com idades até 29 anos e de uma dose para pessoas até 59 anos.^1^

O sarampo persiste entre as principais causas de morte entre crianças, principalmente pela elevada transmissibilidade^2^ com epidemias que chegaram a causar cerca de 2,6 milhões de mortes ao ano no mundo, afetando principalmente crianças ≤ 5 anos.^2^

Em 2016, após cinco anos sem registro de casos, foi concedida ao Brasil a certificação de eliminação do sarampo, entretanto, devido a baixas coberturas vacinais, houve reintrodução do vírus, com surtos em diversos estados.^2^ O cenário de transmissão sustentada no país levou à perda da certificação em 2019^3-5^ e permitiu a identificação de falhas nos programas de imunizações da Atenção Primária à Saúde (APS).^6^

Ainda em 2020, a eliminação da rubéola, doença imunoprevenível e sua principal complicação, a síndrome da rubéola congênita, persiste como meta a ser alcançada, em contextos de baixas coberturas vacinais (70,0%) no mundo.^7^ A Região das Américas, assim como o Brasil, foi declarada livre da rubéola em 2015 pela Organização Mundial da Saúde (OMS).^8^ No Brasil, a certificação da eliminação da rubéola foi alcançada a partir da implantação de estratégias com adoção da vacina contra SCR em campanha nacional de vacinação, e em campanhas de seguimento para crianças e mulheres em idade fértil. Manter a eliminação da rubéola apresenta-se como desafio diante das baixas coberturas vacinais no Brasil.^9^

A reemergência de outras doenças imunopreveníveis, em contextos de redução de cobertura vacinal, vem sendo registrada em vários países, com intensificação no curso da pandemia de covid-19, especialmente pelo aumento das iniquidades sociais e de restrições de acesso à saúde.^4^

Estudo ecológico nacional analisando a cobertura vacinal da primeira dose da vacina contra SCR de 2006-2020 no Brasil, segundo o Índice Brasileiro de Privação de municípios, destacou diminuição generalizada da cobertura vacinal, principalmente entre pessoas mais vulneráveis socialmente, com maior declínio interanual no Norte e Nordeste,^10^ achado semelhante ao verificado em 2021 no Projeto pela Reconquista das Altas Coberturas Vacinais (PRCV).^11^

O risco de reintrodução do sarampo e outras doenças imunopreveníveis com ocorrência de surtos e epidemias no País tem sido apontado por vários estudos, caso medidas de controle consistentes não sejam adotadas para alcance das metas de cobertura vacinal preconizadas pelo PNI, em particular nas regiões com maior desigualdade socioeconômica e de acesso à saúde, como a Nordeste.^3,4,10,11^ Nessa perspectiva, objetiva-se analisar a cobertura vacinal contra SCR em crianças até 24 meses de idade e os fatores associados à não vacinação em coorte de nascidos vivos em 2017 e 2018, residentes em capitais e municípios de grande porte populacional do interior do Nordeste brasileiro. Busca-se reconhecer aspectos centrais para qualificar a gestão e o planejamento em saúde.

## MÉTODOS

### Desenho da pesquisa

Trata-se de inquérito de base populacional realizado de setembro de 2020 a março de 2022 tendo como referência coorte de nascidos vivos em 2017 e 2018 para identificação do percurso de vacinação de crianças de 24 meses de idade. Esta pesquisa representa um recorte do *Inquérito de cobertura vacinal nas capitais de 26 Estados, no Distrito Federal e em 12 municípios do interior em crianças nascidas vivas em 2017−2018 residentes em área urbana*. O delineamento seguiu os procedimentos metodológicos da OMS para compor estimativas de cobertura vacinal.^12^

### Local do estudo

Os locais selecionados do estudo incluíam quatro municípios de grande porte populacional localizados no interior do Nordeste (Vitória da Conquista [Bahia], Caruaru [Pernambuco], Sobral [Ceará] e Imperatriz [Maranhão]) e por nove capitais (São Luís [Maranhão-MA], Teresina [Piauí], Fortaleza [Ceará], Natal [Rio Grande do Norte], João Pessoa [Paraíba], Recife [Pernambuco], Maceió [Alagoas], Aracaju [Sergipe], Salvador [Bahia]).

Constituindo a segunda maior proporção da população brasileira, a região Nordeste com 1.552.175 km², tinha 54.657.621 habitantes em 2022 (26,9% da população do país), com densidade demográfica de 35,21 habitantes/km² e uma proporção de 6,7% (3.635.333) pessoas entre 0 e 4 anos de idade.^13^

### População

A população-alvo foi composta por crianças nascidas vivas em 2017-2018 e residentes nos municípios pesquisados, tendo como fonte de dados o Sistema de Informação de Nascidos Vivos, contendo dados nominais da criança, filiação e residência.

### Procedimento amostral

O processo de amostragem complexa por conglomerados foi constituído a partir dos dados de residência (municípios do interior e capitais) e da utilização de informações sobre a escolaridade/alfabetização, a renda média de responsáveis pelos domicílios e a proporção desses com 20 salários mínimos ou mais, classificando, a partir desses indicadores, os setores censitários em quatro estratos socioeconômicos: A (alto) e B (médio alto), representando áreas com maior renda e alfabetização, e o seu inverso, indicado pelos estratos C (médio baixo) e D (baixo).^12^

A amostra do estudo foi constituída em 3 etapas:

- Etapa 1: composição de estratos socioeconômicos com base sobre escolaridade e renda da pessoa chefe da família, a partir de dados do censo demográfico de 2010 e classificados por setores censitários (A; B; C; D).13
- Etapa 2: composição de conglomerados com ≥ 56 crianças a partir do georreferenciamento dos endereços das crianças nascidos vivos nos setores censitários.
- Etapa 3: seleção por sorteio para cada estrato socioeconômico, de um número variado de conglomerados.

### Coleta de dados e variáveis

A equipe de entrevistadores selecionada realizou capacitação específica e teve acompanhamento pela equipe de pesquisadores nessa etapa de pesquisa.^12^ No processo de trabalho de campo, durante a coleta de dados nos domicílios, foram utilizadas as normas de precaução para a prevenção da transmissão da covid-19.

Nas visitas domiciliares foi utilizado questionário para entrevista com mães/responsáveis pelas crianças para identificação das características sociodemográficas: estratos socioeconômicos (A, B, C, D), município (capital, interior), possui caderneta (sim, não), uso de serviço privado (sim, não), Programa Bolsa Família (PBF) (sim, não), renda familiar (≤ 1.000,00; 1.001,00-3.000,00; 3.001,00-8.000,00; ≥ 8.001,00), escolaridade da mãe em anos de estudo (0-8, 9-12, 13-15, ≥ 16), faixa etária em anos (< 20, 20-34, ≥ 35), trabalho remunerado (sim, não), média de filhos vivos por mãe (variável contínua), sexo da criança (masculino, feminino) e frequência à creche/escola (sim, não). As cadernetas de vacinação das crianças foram fotografadas para avaliação do esquema vacinal básico, incluindo o registro de doses aplicadas no setor privado de vacinação, considerado nesse último caso a realização de pelo menos uma dose de qualquer vacina nesse tipo de serviço.^12^

### Análise

Foram utilizados pesos amostrais para cada domicílio que foi abordado pelo cálculo de probabilidade de seleção, com calibração de acordo com os grupos de populações.^12^

Foi avaliada a cobertura vacinal contra SCR e a evolução desse indicador, incluindo a primeira e a segunda doses válidas recebidas pelas crianças até os 24 meses de idade, considerando-se completude com duas doses, como preconizado pelo PNI. Dessa forma, considerou-se para o cálculo da cobertura vacinal as últimas doses válidas do esquema completo, em relação ao total de nascidos vivos. Com base na composição das vacinas utilizadas para proteger as mesmas doenças nos cenários da rede pública e privada, procedeu-se à junção das datas das vacinas contra SCR e tetraviral (contra SCR e varicela - SCRV), levando-se em consideração a faixa etária equivalente para cada dose aplicada para o cálculo da cobertura vacinal. Foram definidas como válidas as primeiras doses da vacina contra SCR aplicadas acima de 365 dias de idade e as segundas doses aplicadas pelo menos após 30 dias da primeira dose.^1,12,14^

Estimativas ponderadas de coberturas vacinais com os pesos e os respectivos intervalos de confiança (IC_95_%) foram calculados para as duas doses, para cada dose e para o esquema completo contra SCR, segundo estratos socioeconômicos e municípios (capitais e interiores). O critério para significância estatística foi baseado no valor de p < 0,05.^12^

Para a análise dos fatores de risco para vacinação incompleta contra SCR (crianças que não tomaram todas as doses) incorporou-se nos modelos a razão de chance ajustada (*Odds ratio*: OR-a) com respectivos IC_95_%, previstos para a regressão logística. No modelo de regressão logística simples, as categorias de variáveis com valor de p < 0,20 foram incluídas no modelo de análise pelo método *stepwise*, verificando-se o efeito conjunto independente para a ocorrência de vacinação incompleta e a existência de colinearidade entre variáveis pela análise do fator de inflação da variância. A variável dependente, *status* de vacinação, levou em consideração as duas doses válidas da vacina contra SCR, tendo sido dicotomizada em vacinação incompleta (“incompletude vacinal”) ou vacinação completa (“esquema completo de doses”). 

Foram definidas três categorias para as doses válidas:

- Sem registro de doses (criança não tomou nenhuma dose da vacina)
- Esquema incompleto de doses (criança tomou uma dose da vacina)
- Esquema completo de doses (criança tomou as duas doses da vacina)

O status de vacinação incompleto foi composto pelas categorias “Sem registro de doses” e “Esquema incompleto de doses”. Para vacinação em conformidade considerou-se a categoria “Esquema completo de doses”.

Também foi avaliada a taxa de abandono da segunda dose em relação à primeira dose ([percentual de primeira dose – percentual de segunda dose]/percentual de primeira dose).

Os dados foram apresentados segundo estrato socioeconômico, capitais e municípios do interior. Para a análise estatística utilizou-se o STATA versão 17 (StataCorp LLC, College Station, TX).

### Considerações éticas

A pesquisa foi aprovada pelos Comitês de Ética em Pesquisa com Seres Humanos do Instituto de Saúde Coletiva da Universidade Federal da Bahia, sob parecer número 3.366.818, em 04 de junho de 2019, com Certificado de Apresentação de Apreciação Ética (CAAE) 4306919.5.0000.5030; e da Irmandade da Santa Casa de São Paulo, sob parecer número 4.380.019, em 04 de novembro de 2020, com CAAE 39412020.0.0000.5479.

## RESULTADOS

Na avaliação da amostra de 12.137 nascidos vivos em 2017-2018 (Tabela 1), a maior cobertura vacinal foi verificada na primeira dose (89,7%, IC_95_% 87,6;91,4), principalmente no estrato C (92,7%, IC_95_% 90,7;94,4). Entre as capitais, a melhor cobertura vacinal (completa) foi verificada em Teresina (90,9%, IC_95_% 85,9;94,3), enquanto Natal teve a pior cobertura vacinal (67,1%, IC_95_% 53,7;78,2). Vitória da Conquista apresentou a menor cobertura vacinal completa entre os municípios do interior (74,0%, IC_95_% 63,4;82,5) (Figura 1). Nas capitais estimou-se perda amostral de 525 nascidos vivos (4,8%)^13^ e nos municípios do interior não houve perdas.

**Figura 1:** Cobertura vacinal da primeira e segunda doses, e cobertura completa da vacina contra sarampo, caxumba e rubéola em uma coorte de nascidos vivos em 2017-2018 em capitais e municípios de interior do Nordeste do Brasil, por estrato socioeconômico (n = 12.137)

**Tabela 1:** Frequência absoluta e relativa de nascidos vivos em 2017 e 2018 em capitais e municípios de grande porte do interior do Nordeste do Brasil, segundo estratos socioeconômicos (n = 12.137)

Variáveis/estratos socioeconômicos	A	B	C	D	Total
	**n (%)**	**n (%)**	**n (%)**	**n (%)**	**n (%)**
**Total**	2.701 (22,3)	3.118 (25,7)	3.145 (25,9)	3.173 (26,1)	12.137 (100,0)
**Capitais**					
São Luís	182 (6,7)	223 (7,2)	224 (7,1)	225 (7,1)	854 (7,0)
Teresina	227 (8,4)	225 (7,2)	222 (7,1)	225 (7,1)	899 (7,4)
Fortaleza	312 (11,6)	432 (13,9)	423 (13,4)	445 (14,0)	1.612 (13,3)
Natal	84 (3,1)	153 (4,9)	223 (7,1)	225 (7,1)	685 (5,6)
João Pessoa	226 (8,4)	225 (7,2)	226 (7,2)	227 (7,2)	904 (7,4)
Recife	330 (12,2)	447 (14,3)	462 (14,7)	450 (14,2)	1.689 (13,9)
Maceió	205 (7,6)	279 (8,9)	219 (7,0)	226 (7,1)	929 (7,7)
Aracaju	233 (8,6)	219 (7,0)	222 (7,1)	226 (7,1)	900 (7,4)
Salvador	450 (16,7)	456 (14,6)	456 (14,5)	456 (14,4)	1.818 (15,0)
**Municípios de Interior**					
Imperatriz	120 (4,4)	113 (3,6)	118 (3,8)	114 (3,6)	465 (3,8)
Sobral	103 (3,8)	119 (3,8)	120 (3,8)	123 (3,9)	465 (3,8)
Caruaru	113 (4,2)	114 (3,7)	116 (3,7)	119 (3,8)	462 (3,8)
Vitória da Conquista	116 (4,3)	113 (3,6)	114 (3,6)	112 (3,5)	455 (3,7)

Nos domicílios, o benefício do PBF foi citado por 36,0% das mães/responsáveis, com maior frequência no estrato D (49,9%). A renda familiar de ≤ R$ 1.000,00 foi relatada por 38,0% das famílias (Tabela 2).

**Tabela 2:** Características sociodemográficas em (%) com intervalos de confiança de 95% (IC_95%_), da família, da mãe e da criança, em uma coorte de nascidos vivos em 2017 e 2018 em capitais e municípios de grande porte do interior do Nordeste do Brasil, segundo estratos socioeconômicos (n = 12.137)

Variáveis/Estratos socioeconômicos	A	B	C	D	Total
	**% (IC**_95_ **%** ^b^ **)**	**% (IC**_95_ **%** **)**	**% (IC**_95_ **%** **)**	**% (IC**_95_ **%** **)**	**% (IC**_95_ **%** **)**
**Características da família**					
**Programa Bolsa Família (sim)**	7,9 (5,7;11,0)	20,1 (15,7;25,4)	37,8 (33,7;42,1)	49,9 (45,5;54,3)	36,0 (33,4;38,7)
**Renda familiar mensal (R$)**					
≤ 1.000,00	5,7 (3,8;8,3)	19,1 (15,3;23,6)	37,3 (32,5;42,2)	55,5 (51,1;59,9)	38,0 (35,0;41,2)
1.001,00-3.000,00	12,1 (8,6;16,6)	30,8 (25,5;36,7)	44,1 (39,4;49,0)	35,0 (30,5;39,8)	32,5 (29,7;35,5)
3.001,00-8.000,00	27,5 (20,6;35,8)	24,6 (19,7;30,4)	14,8 (10,4;20,6)	3,4 (2,5;4,7)	12,9 (10,9;15,3)
≥ 8.001,00	35,3 (26,3;45,5)	11,9 (6,0;22,2)	1,1 (0,7;1,8)	0,3 (0,0;0,9)	8,0 (5,9;10,8)
Não sabe/não respondeu	19,4 (11,1;31,9)	13,6 (8,3;21,5)	2,8 (1,9;4,1)	5,8 (3,6;9,3)	8,5 (6,3;11,4)
**Características da mãe**					
**Faixa etária ao nascimento da criança (anos)**					
< 20	1,0 (0,6;1,9)	1,1 (0,7;1,8)	2,5 (1,8;3,6)	4,5 (3,3;6,2)	3,0 (2,4;3,9)
20-34	44,8 (37;52,8)	50,0 (44,2;55,9)	67,9 (64,5;71,2)	65,1 (61,3;68,7)	60,2 (57,5;62,8)
≥ 35	53,9 (45,8;61,8)	48,3 (42,2;54,4)	29,3 (26,1;32,6)	30,0 (26,1;34,3)	36,4 (33,6;39,3)
Não sabe/não respondeu	0,3 (0,2;0,6)	0,6 (0,3;1,0)	0,3 (0,0;0,8)	0,4 (0,2;1,0)	0,4 (0,2;0,6)
**Escolaridade (anos)**					
0-8	2,1 (1,3;3,1)	6,3 (4,6;8,7)	8,3 (6,5;10,5)	15,8 (13,4;18,6)	10,5 (9,3;11,9)
9-12	4,8 (2,9;7,7)	9,9 (7,5;12,9)	18,0 (14,8;21,8)	22,1 (19;25,6)	16,6 (14,8;18,5)
13-15	28,0 (21,9;35,1)	33,0 (26,4;40,2)	54,1 (50,3;57,9)	49,4 (45,2;53,5)	44,5 (41,7;47,4)
≥ 16	61,9 (54,2;69,0)	47,6 (38,7;56,6)	17,1 (14,1;20,6)	10,1 (5,5;18,0)	25,6 (22,0;29,7)
Não sabe/não respondeu	3,3 (1,6;7,1)	3,3 (1,8;5,8)	2,5 (1,6;3,8)	2,6 (1,8;3,7)	2,8 (2,2;3,6)
**Trabalho remunerado (sim)**	68,6 (60,8;75,4)	56,2 (50,8;61,4)	43,0 (39,5;46,5)	36,4 (33,0;39,9)	46,0 (43,5;48,7)
**Média de filhos vivos por mãe**	1,91 (1,87;1,95)	1,99 (1,95;2,03)	2,03 (1,99;2,07)	2,21 (2,17;2,26)	2,04 (2,02;2,06)
**Características da criança**					
**Sexo da criança**					
Masculino	50,4 (43,0;57,7)	51,4 (46,6;56,1)	51,6 (47,5;55,7)	50,7 (47,8;53,6)	50,9 (48,8;53,1)
Feminino	49,6 (42,3;57,0)	48,6 (43,9;53,4)	48,4 (44,3;52,5)	49,3 (46,4;52,2)	49,1 (46,9;51,2)
**Possui caderneta (sim)**	98,9 (95,9;99,7)	99,3 (98,7;99,6)	99,1 (98,2;99,6)	99,0 (97,2;99,6)	99,0 (98,3;99,4)
**Uso de serviço privado para a vacinação (sim)**	52,2 (43;61,3)	26,0 (19,1;34,3)	7,8 (6,0;10,1)	5,7 (2,2;13,8)	16,9 (13,6;20,8)
**Frequenta creche/escola (sim)**	48,7 (37,9;59,6)	37,6 (29,9;45,9)	34,4 (29,8;39,4)	31,1 (26,7;35,8)	35,7 (32,4;39,1)

As mães das crianças pesquisadas encontravam-se em maior proporção na faixa etária entre 20 e 34 anos (60,2%), com escolaridade entre 13 e 15 anos de estudo (44,5%) e trabalho remunerado (46,0%), com média de filhos(as) por mãe de 2,04 (Tabela 2).

As crianças apresentaram maior frequência para o sexo masculino (50,9%), a caderneta de vacinação foi encontrada em 99,0% da amostra, sem diferença entre os estratos. Verificou-se a utilização de serviço privado de vacinação por 16,9% das crianças, com maior frequência no estrato A (52,2%), e presença de creche/escola em 35,7% da amostra, com menor frequência no estrato D (31,1%) (Tabela 2).

Identificou-se associação entre a não vacinação contra SCR, considerando o esquema vacinal completo, com as seguintes características sociodemográficas: residência em áreas correspondentes ao estrato A (OR-a 1,29, IC_95%_ 1,10;1,50), residência no interior (OR-a 1,22, IC_95%_ 1,07;1,39), não acesso ao PBF (OR-a 1,19, IC_95%_ 1,05;1,34), renda familiar ≤ R$ 1.000,00 (OR-a 1,17, IC_95%_ 1,03;1,31), mãe não ter trabalho remunerado (OR-a 1,28, IC_95%_ 1,15;1,42), ter mais de um filho vivo por mãe (OR-a 1,12, IC_95%_ 1,08;1,17) e ausência de caderneta de vacinação (OR-a 10,69, IC_95%_ 6,27;18,20) (Tabela 3).

**Tabela 3:** Razão de chances (odds ratio, OR) bruta e ajustada e intervalo de confiança de 95% (IC_95%_) da vacinação incompleta e não vacinação contra sarampo, caxumba e rubéola pelos fatores sociodemográficos de crianças nascidas em 2017 e 2018, residentes em capitais e municípios de grande porte populacional do interior do Nordeste do Brasil (n=12.137)

Variáveis	OR bruta (IC_95%_)	p-valor	OR ajustada (IC_95%_)	p-valor
Estratos socioeconômicos				
A	1,42 (1,25;1,61)	0,138	1,29 (1,10;1,50)	0,057
B	1,35 (1,19;1,53)		1,27 (1,11;1,46)	
C	1,00		1,00	
D	1,19 (1,05;1,35)		1,15 (1,00;1,32)	
**Município**				
Capital	1,00	0,012	1,00	0,001
Interior	1,17 (1,03;1,31)		1,22 (1,07;1,39)	
**Características da família**				
**Programa Bolsa Família**				
Sim	1,00	< 0,001	1,00	0,014
Não	1,20 (1,10;1,32)		1,19 (1,05;1,34)	
**Renda familiar mensal (R$)**				
≤ 1.000,00	1,06 (0,95;1,18)	0,026	1,17 (1,03;1,31)	0,300
1.001,00-3.000,00	1,00		1,00	
3.001,00-8.000,00	1,15 (1,00;1,32)		1,03 (0,87;1,21)	
≥ 8.001,00	1,15 (0,97;1,37)		0,92 (0,73;1,15)	
**Características da mãe**				
**Faixa etária ao nascimento da criança (anos)**				
< 20	1,00	0,860	–	–
20-34	1,30 (0,98;1,73)		–	
≥ 35	1,26 (0,95;1,68)		–	
**Escolaridade (anos)**				
0-8	1,00	0,571	–	–
9-12	1,10 (0,93;1,29)		–	
13-15	0,99 (0,85;1,14)		–	
≥ 16	1,10 (0,94;1,28)		–	
**Trabalho remunerado da mãe**				
Sim	1,00	< 0,001	1,00	< 0,001
Não	1,27 (1,16;1,39)		1,28 (1,15;1,42)	
**Filhos vivos**				
**Média de mais de um filho vivo por mãe**	1,08 (1,04;1,12)	< 0,001	1,12 (1,08;1,17)	< 0,001
**Características da criança**				
**Sexo da criança**				
Masculino	1,00	0,278	–	–
Feminino	1,05 (0,96;1,15)		–	
**Possui caderneta**				
Sim	1,00	< 0,001	1,00	< 0,001
Não	10,93 (6,96;17,15)		10,69 (6,27;18,20)	
**Uso de serviço privado para a vacinação**				
Sim	1,24 (1,10;1,40)	0,001	1,12 (0,95;1,33)	0,310
Não	1,00		1,00	
**Frequenta creche/escola**				
Sim	1,00	0,021	1,00	0,179
Não	1,11 (1,02;1,22)		1,08 (0,97;1,19)	

Na análise de padrões de completude das doses (sem registro de doses, esquema de doses incompleto e esquema de doses completo), a cobertura vacinal completa com duas doses válidas contra SCR foi de 79,3% (IC_95_% 76,5;81,8). Verificou-se maior e menor proporção, respectivamente, nos estratos C (83,0%, IC_95%_ 79,9;85,7) e A (70,9%, IC_95%_ 59,3;80,2). Para 9,5% (IC_95%_ 7,8;11,4) das crianças (n = 1.179) não houve registro de nenhuma dose contra SCR e 11,3% (IC_95%_ 9,8;12,9) das crianças (n = 1.365) não completaram o esquema vacinal. No estrato A verificou-se a maior proporção de crianças sem registro de doses aplicadas (14,9%, IC_95%_ 9,8;22,1) e com esquema de doses incompleto (14,2%, IC_95%_ 9,2;21,3). Para as capitais, Natal apresentou a maior proporção de crianças sem doses aplicadas (15,9%, IC_95%_ 10,4;23,7) como também de doses incompletas (16,9%, IC_95%_ 10,8;25,7). Nos municípios do interior, Vitória da Conquista destacou-se com a maior proporção de crianças sem registro de doses aplicadas contra SCR (15,2%, IC_95%_ 6,9;30,3) (Tabela 4).

**Tabela 4:** Proporção (%) e intervalos de confiança de 95% (IC_95%_), de não vacinação, vacinação incompleta, cobertura da vacina contra sarampo, caxumba e rubéola, e taxa de abandono em uma coorte de nascidos vivos de 2017 e 2018 em capitais e municípios de grande porte populacional do interior do Nordeste, Brasil, por estrato socioeconômico (n = 12.137)

Variáveis	Não vacinação (sem registro de doses)	Vacinação incompleta (esquema incompleto de doses)	Cobertura vacinal (esquema completo de doses)	Taxa de abandono
	**% (IC**_95_ **%** **)**	**% (IC**_95_ **%** **)**	**% (IC**_95_ **%** **)**	**%**
**Total**	9,5 (7,8;11,4)	11,3 (9,8;12,9)	79,3 (76,5;81,8)	10,6
**Estratos socioeconômicos**				
A	14,9 (9,8;22,1)	14,2 (9,2;21,3)	70,9 (59,3;80,2)	15,0
B	10,0 (6,8;14,5)	10,6 (8,0;13,9)	79,4 (72,8;84,8)	10,9
C	6,6 (5,1;8,6)	10,4 (8,3;13,0)	83,0 (79,9;85,7)	9,8
D	8,7 (6,4;11,7)	10,8 (9,1;12,8)	80,6 (77,2;83,5)	9,4
**Capitais**				
São Luís	7,6 (4,1;13,7)	16,7 (11,7;23,2)	75,7 (67,5;82,4)	10,6
Teresina	2,9 (1,4;6,1)	6,2 (3,4;10,8)	90,9 (85,9;94,3)	5,9
Fortaleza	11,9 (8,1;17,2)	13,0 (9,1;18,2)	75,1 (65,9;82,5)	13,2
Natal	15,9 (10,4;23,7)	16,9 (10,8;25,7)	67,1 (53,7;78,2)	19,0
João Pessoa	10,5 (6,5;16,6)	14,4 (11,5;17,7)	75,1 (68,2;80,9)	14,7
Recife	7,9 (4,1;14,7)	9,1 (6,9;11,9)	83,0 (77,2;87,6)	9,5
Maceió	15,9 (7,7;30,0)	9,4 (6,4;13,6)	74,6 (64,1;82,9)	8,6
Aracaju	11,6 (6,8;19,3)	9,5 (5,3;16,3)	78,9 (69,2;86,2)	8,0
Salvador	5,4 (3,7;7,9)	8,6 (5,9;12,4)	86,0 (81,6;89,5)	7,1
Capitais	9,6 (7,8;11,7)	11,3 (9,7;13,0)	79,2 (76,1;81,9)	10,4
**Interiores**				
Imperatriz	6,9 (4,0;11,5)	16,3 (10,2;24,9)	76,8 (69,2;83,1)	17,5
Sobral	8,5 (2,9;22,5)	7,3 (4,0;13,0)	84,3 (67,9;93,1)	7,7
Caruaru	3,4 (1,6;7,1)	10,0 (6,2;15,6)	86,7 (79,0;91,8)	10,2
Vitória da Conquista	15,2 (6,9;30,3)	10,8 (6,6;17,2)	74,0 (63,4;82,5)	12,5
Interior	8,4 (5,4;12,9)	11,3 (8,7;14,6)	80,3 (75,2;84,5)	12,2

A taxa de abandono da segunda dose contra SCR foi de 10,6%, sendo 15,0% no estrato A. Os municípios que apresentaram as maiores proporções de taxa de abandono foram: Natal (19,0%) e João Pessoa (14,7%) entre as capitais, e Imperatriz (17,5%) no interior do Nordeste (Tabela 4).

## DISCUSSÃO

Este estudo confirma o cenário preocupante de baixas coberturas vacinais contra SCR sem alcance do esquema completo de vacinação em mais de um quinto das crianças até 24 meses de idade nas capitais e em quatro municípios de grande porte populacional do interior do Nordeste do Brasil, no período de 2017-2018. Digno de nota, é o fato crítico de 10% da população infantil pesquisada não ter registro de nenhuma dose contra SCR na caderneta de vacinação avaliada. A meta de cobertura vacinal estipulada pelo PNI (95%) não foi alcançada contra SCR em nenhuma capital e município do interior do Nordeste,^15,16^ com menores coberturas vacinais para Natal e Vitória da Conquista, respectivamente. Crianças residentes em áreas correspondentes ao estrato socioeconômico mais alto e em municípios do interior, com renda familiar ≤ R$ 1.000,00, ausência da caderneta de vacinação, mães sem trabalho remunerado, com mais de um filho, não frequentando escola/creche e sem acesso ao PBF foram condições associadas à vacinação incompleta e/ou à não vacinação contra SCR em municípios do Nordeste do Brasil. A suscetibilidade ampliada de parte considerável das crianças dessas coortes para sarampo, estende-se também para caxumba e rubéola.

Trata-se de cenário que traduz a ampliação de suscetibilidade e manutenção de risco de transmissão de doenças imunopreveníveis^17^ e reitera as diferentes dimensões de vulnerabilidade social, demandando, portanto, estratégias inovadoras mais ativas para intensificação das ações de vacinação no Sistema Único de Saúde (SUS).^10^

A associação entre vacinação incompleta e/ou não vacinação contra SCR e residência em municípios de grande porte populacional do interior do Nordeste revela, de forma ainda mais contundente, possíveis barreiras de acesso na APS além de iniquidades sociais e de saúde.^10^ Ressalta-se que o não alcance das ações na APS pela garantia de cobertura populacional esteve associado à vacinação incompleta na região Nordeste do Brasil em diferentes estudos.^4,18,19^

Essas falhas de saúde pública em municípios do interior incluem questões relativas a contextos rurais^13^ onde ainda persistem problemas importantes para alcance de cobertura e qualidade na vacinação em geral.^18^ A cobertura vacinal nessas realidades rurais foi verificada como sendo cerca de 1,5 vez menor do que em áreas urbanas. Da mesma forma, residir em estratos com melhores condições socioeconômicas nas capitais e nos municípios do interior pesquisados no Nordeste esteve associado significativamente à vacinação incompleta e/ou não vacinação contra SCR. Esse achado possivelmente está associado a questões relacionadas à hesitação vacinal, demonstrada pela decisão dos pais/responsáveis adiarem e/ou recusarem a vacinar seus filhos(as),^19^ fato mais comumente verificado nessas populações.^14^ Outra possível explicação é o maior acesso aos serviços privados de vacinação, que pode ter contribuído com a não conclusão do esquema completo, possivelmente em virtude da inadequação do seguimento acerca da situação vacinal pelo serviço público de saúde, além da comunicação com serviços privados sobre estratégias e dados oportunos de vacinação.^19^

As baixas coberturas contra SCR evidenciadas previamente por outros inquéritos nacionais no Brasil^19^ persistem atualmente, agravadas pelas elevadas taxas de abandono entre municípios do interior e capitais do Nordeste,^14^ fato que contribui para o risco de reintrodução e/ou recorrência de casos, principalmente entre populações com maior vulnerabilidade social habitualmente mais acometidas por doenças imunopreveníveis e com síndromes clínicas mais graves.^16,18^

Embora estudos demonstrem o declínio das coberturas vacinais com ocorrência de forma global no Brasil, com alcance da meta preconizada pelo PNI apenas por metade dos municípios, disparidades socioeconômicas retratadas na região Nordeste apontam para a ocorrência dessa problemática de forma ainda mais acentuada, demonstrada para além de menores coberturas vacinais, pelas maiores reduções anuais da adoção da vacina contra SCR.^10^

Nos anos de 2015 e 2016, a região das Américas foi declarada como área livre de transmissão da rubéola e do sarampo. No entanto, no ano da coorte mais recente (2018), nas regiões Norte e Nordeste, houve, respectivamente, a reintrodução do vírus do sarampo e maior incidência da doença no Brasil,^20^ contexto epidemiológico crítico diante de baixas e heterogêneas coberturas contra SCR no país^4,21,22^ e ao alto fluxo migratório entre países com transmissão da doença, na América Latina. A certificação da eliminação do sarampo no Brasil foi perdida em 2019, após um ano de transmissão contínua da doença,^4^ sendo que a manutenção da eliminação da rubéola^7,8^ segue como desafio nesse cenário de baixas coberturas vacinais.^14^

As iniquidades em saúde intensificadas pela pandemia de covid-19 em 2020,^23^ além da redução de acesso a serviços de saúde no SUS, contribuíram para a diminuição das ações de vacinação, fatos que, em conjunto, justificam a manutenção e até mesmo o agravamento das baixas coberturas vacinais contra o sarampo, mas também caxumba e rubéola, no Brasil.^4,24^

Nesses cenários, é imperativo destacar a importante atuação da APS como porta de entrada e coordenadora do cuidado no âmbito do SUS para a promoção de ações de vacinação, avaliação, monitoramento e seguimento domiciliar ativo para garantia de manutenção do calendário vacinal atualizado. O monitoramento e a avaliação das ações de vacinação em desenvolvimento, bem como a divulgação de informações sobre a situação vacinal, são medidas estratégicas para a tomada de decisão na vigilância das coberturas vacinais, além de serem importantes evidências para direcionar o alcance dos objetivos do PNI.^25^

A redução nas coberturas vacinais contra SCR na última década manteve o risco elevado para a recorrência de casos no país, mesmo sendo ação fundamental para controle. Ações de informação, educação e comunicação, a qualificação da vigilância integrada à APS, o reconhecimento de barreiras de acesso para equidade vacinal e o fortalecimento do PNI com ampliação de disponibilidade de vacinas e profissionais de saúde são essenciais para a recuperação de altas coberturas vacinais e sua homogeneidade.^4,19,21,26^

Benefício mais comum entre famílias do estrato com piores condições socioeconômicas, o PBF representa uma importante política de transferência de renda no Brasil, contribuindo decisivamente para maior adesão à vacinação. Trata-se de condicionante estratégico, uma vez que requer a atualização do calendário vacinal completo para a manutenção do benefício e, dessa forma eficaz, medida para adequação das coberturas vacinais, inclusive contra SCR.^27^

Por outro lado, o planejamento estratégico nos territórios é fundamental e custo-efetivo, mesmo diante da alta complexidade e custo para a execução em diferentes realidades brasileiras.^24^ As ações devem trazer em perspectiva também abordagens mais eficientes, com foco nos desafios locais, alinhadas com a Agenda de Imunização 2030,^6^ a exemplo do PRCV, desenvolvido de forma pactuada e compartilhada, a partir dos sistemas locais de saúde, nos territórios da APS, com ampla participação social.^11^

As limitações deste estudo compreendem o uso de dados do censo demográfico de 2010 para a estratificação e seleção dos setores censitários e constituição da amostra em cada estado pesquisado. A restrição do acesso de pesquisadores(as) aos domicílios, principalmente nos estratos socioeconômicos mais altos e em virtude da ocorrência da pandemia por covid-19, gerou limitações para a coleta de dados em alguns domicílios, particularmente em decorrência do medo da população relacionada à transmissão da doença. A baixa qualidade das imagens de algumas cadernetas de vacinação, além da ausência de padrão para os registros de doses e vacinas aplicadas pelos serviços públicos e privados de vacinação, pode ter gerado erros no registro de dados da caderneta de vacinação. Para minimizar esses erros foram realizados treinamentos e supervisões à equipe de coleta de dados e de digitação das informações coletadas das cadernetas de vacinação. Mesmo com essas limitações, este estudo traz importantes evidências para melhor compreensão a respeito do cenário crítico de baixas coberturas vacinais para o sarampo e, dessa forma, risco aumentado da transmissão de doenças imunopreveníveis, incluindo também caxumba e rubéola. Ressalta-se a importância de se ter maior compreensão das limitações, relacionadas às condições socioeconômicas e de acesso aos serviços de saúde, para a implementação de estratégias ainda mais eficientes e factíveis de ampliação do acesso às ações de vacinação nos territórios da APS.

Em conclusão, confirmam-se baixas coberturas vacinais e o elevado percentual de não vacinação contra SCR na população infantil de 24 meses de idade de capitais e municípios do interior do Nordeste. Ressaltam-se as baixas coberturas vacinais completas do esquema contra SCR, principalmente para a segunda dose. O declínio desse percentual na coorte de nascidos vivos de 2017-2018 reforça a persistência e ampliação do problema, além de um crítico cenário de não vacinação, com mais de mil crianças sem nenhuma dose de vacinação contra SCR, em diferentes contextos de iniquidades sociais.
